# The Hospital. Nursing Section

**Published:** 1904-07-23

**Authors:** 


### The Hospital

**Nursing Section.**

Contributions for this Section of "The Hospital" should be addressed to the Editor, "The Hospital" Nursing Section, 28 & 29 Southampton Street, Strand, London, W.C.

No. 930 —Vol. XXXVI. *SATURDAY, JULY* 23, 1904.

### Notes on News from the Nursing World.

### QUEEN ALEXANDRA AND HER NURSES.

The reception by Queen Alexandra at Buckingham Palace, on Friday, the 22nd instant, of Pension Fund Nurses, to whom she will present certificates, possesses a special interest for women workers everywhere. It is a fact, which few remember, that Florence Nightingale, and the majority of those who employed or trained nurses, held in 1887, when the Fund was founded, that nurses could not save anything out of their limited earnings. Their Majesties the King and Queen consistently took a different view, and to their continuous support and confidence the Royal National Pension Fund owes most of its success. In about 16 years the nurses have paid in nearly £1,000,000, and to-day the Pension Fund is the greatest thrift agency for women workers in the world. We hope that both the King and the Queen may realise the inestimable service which they have rendered by their courageous support of the Pension Fund. To-day it is the wonder and the pride of the nursing world.

### QUEEN ALEXANDRA'S MILITARY NURSING SERVICE.

We are officially informed that Miss L. M. Toller, and Miss L. F. A Waller have been appointed staff nurses in Queen Alexandra's Imperial Military Nursing Service. Miss Toller has been posted to Portsmouth and Miss Waller to Connaught Hospital, Aldershot. Miss H. W. Reid, matron, has been transferred from Aldershot to Wynberg, South Africa; and Miss E. A. Wilkinson, matron, from Woolwich to Bloemfontein, South Africa. Sister C. Anderson has been transferred from Cape Town to Pretoria: Sister M. E. Harper, from Shorncliffe to Harrismith; Sister M. Wright, from Cork to Bloemfontein; Sister E. H. Hordley, from Wynberg to Pretoria, as secretary to the principal matron's staff. Staff nurse K. M. Hewetson has been transferred from the Royal Military College, Sandhurst, to Alton, for temporary duty. The appointments of Sisters F. M. Hodgins and E. H. Hordley, and of Staff Nurses K. M. Hewetson, A. E. Fitzgerald, S. K. Bills, and B. N. Daker have been confirmed.

### NURSING PRINCESS VICTORIA OF SCHLESWIG-HOLSTEIN.

In accordance with the specially expressed wish of Princess Victoria of Schleswig-Holstein, eldest daughter of Prince and Princess Christian, the operation for appendicitis which she had to undergo a few days since was performed at the Prince Christian Victor Memorial Home at Windsor. The Princess has been attended from the first by the nurses attached to the Home, who are known as "Princess Christian Trained Nurses." They naturally appreciate the compliment paid to them by her Highness who desired to be under their care. Princess Christian has paid several visits to her daughter, who is now, happily, reported convalescent.

### A SURPRISE ROYAL VISIT.

The matron of the Hospital and Home for Incurable Children had the honour on Friday afternoon of receiving the Duchess of Saxe-Coburg and Gotha and Princess Henry of Battenberg, who paid a surprise visit to the institution at 2 Maida Vale, after visiting the Hospital for Epilepsy and Paralysis next door. They first inspected the patients in the garden, afterwards the wards, and finally the children's embroidery, carving, and basket work, and seemed much pleased with all they saw. They were interested in hearing about the new premises at Hampstead, to which the hospital will shortly be moved.

### AMERICAN NURSES AND THE GENERAL SLOCUM DISASTER.

In another column Miss M. Ard Mackenzie, superintendent of the Brooklyn Hospital Training School for Nurses, graphically describes the efforts of the nurses on the occasion of the terrible disaster to the *General Slocum* excursion steamer. It will be noticed that the services of the nurses of no fewer than six hospitals were at once available, and that those who participated in the work were not only called to display their skill in nursing, but also, in a peculiar manner, their power in adapting themselves to circumstances. Dressing the wound of a patient in a hospital ward, in an atmosphere of perfect order, is, obviously, the antithesis of wading into water strewn with the bodies of the dead and dying, laying hold of any in whom life seems to be not extinct, bringing them to land, and then commencing the ordinary functions of a nurse. In such a case as this, a nurse who pins her faith entirely to routine would be of little use.

### THE OLDHAM APPEAL.

It will be remembered that in November last a case was tried at Manchester Assizes before Mr. Justice Jelf and a special jury, the defendants being the Oldham Nursing Association. The sum of £300 was then awarded to a patient who had sustained personal injuries through negligence on the part of two nurses employed by the Association. We approved the verdict at the time on the ground that, if the injuries were incurred, the Association could not escape responsibility for the actions of their nurses. On this particular point the Court of Appeal, consisting of the Master of the Rolls and Lords Justices Stirling and Mathew, to whom the case was remitted, pronounced on Friday in favour of the Association. With all due respect to these eminent judges, we cannot think that their decision, though it may be admirable law, is in accordance with common sense or justice. It cannot be pleaded on behalf of the Oldham Association that they are a purely philanthropic body, for the nurses sent out under the auspices of the committee are salaried servants, and the fees they earn help to pay the general expenses. Yet, according to the judgment of the Court of Appeal, the Association are not in any sense accountable for the actions of their employees, and patients who may be injured in consequence of their incompetence have absolutely no means of obtaining redress. As it cannot be expected that people who can afford fees will care to employ nurses whose principals wash their hands of any legal obligations in respect to them, the reversal of the verdict of the jury at Manchester can scarcely fail to seriously affect the position of all organisations conducted on similar lines to the Oldham Nursing Association.

### GUY'S NURSES AT KINGSWOOD.

LAST week the sisters and nurses of Guy's Hospital were, as usual at this period of the year, entertained by Mr. and Mrs. Cosmo Bonser, at Kingswood. The weather, both on Wednesday and Thursday, was superb, and the Treasurer and his wife spared no effort to promote the enjoyment of their guests.

### THE NURSING QUESTION AT THE CARDIFF SEAMEN'S HOSPITAL.

THE trustees under the will of the late Marquis of Bute appear to be obdurate. In other words, they have declined to contribute any more money to the support of the Cardiff Seamen's Hospital until the question of the nursing is settled. Whatever may happen, we cannot blame the hospital committee, who have decided that they cannot agree that any department of the institution shall be carried on under the management of any religious body or any guild or sisterhood. But they are also willing to appoint a certain number of Roman Catholic nurses to attend to Roman Catholic patients. This, we think, is all that the trustees ought to require. The late Lord Bute, by intimating that he desired his views to be observed "if possible," left the door to compromise open, and those who slam it in the face of the committee expose themselves to the reproach of being less anxious to assist a good cause than to persist in an attitude which suggests intolerance.

### DISGRACEFUL SCANDALS AT A WORKHOUSE INFIRMARY.

SOME extraordinary statements were made in the course of an inquiry by an Inspector of the Local Government Board, into charges preferred against certain officials of the Wallingford Workhouse Infirmary, and against the general administration. It was admitted by the superintendent nurse that she threw a bucket of hot water over the labour master. The labour master admitted that when this happened the other nurses came out and a struggle ensued, so that all of them were on the ground together. He also admitted that on two occasions he went to the infirmary without permission, in distinct contravention of the rules. An assistant nurse admitted that she had seen a child suffering from a skin disease shut out on the balcony in frosty weather as a punishment; a probationer's statement that she had bathed men in the infirmary without either of the other nurses being present, was not challenged; and the inspector recommended to the consideration of the master his observation, "that the evidence went to show that there was considerable laxity in the administration of the place." It appears that the superintendent nurse, an assistant nurse, and the labour master, resigned before the inquiry opened; but we agree with the inspector that their resignations did not render his investigation superfluous, and we await the issue of the report with interest.

### QUEEN'S NURSES AT WOLVERHAMPTON.

THE sixth annual report of the Queen Victoria's Nursing Institute, Wolverhampton, which has just been issued, is especially interesting, as the charity has lately been reorganised. Under the new rules passed by the managing committee the entire town is open to the four nurses who work in four districts. The Committee desire to express their appreciation of the energetic services rendered by the matron, Miss Nicholson, and the thorough work done by the nurses. The matron paid 963 visits in the year ending April 30th, and the nurses 14,107. Upwards of 30 fresh annual subscribers were registered during the year, and it is hoped that the number may still be largely increased. The handsome sum of £45 was realised by the dance in January.

### BANGOR DISTRICT NURSING SOCIETY.

THE honorary secretary of the Bangor District Nursing Society, County Down, wishes to supplement our reference to the report of the annual meeting last week, by the statement that all the speakers at the annual meeting acknowledged Miss Borlase's services in the very warmest manner. She adds: "The Committee hope to make a good use of the balance they have in hand, but it is not possible for the nurse in charge of the country district to relieve Nurse Borlase of any of her work in the town, as you seem to suggest she might do. The distances to be traversed are far too great." This inference is a mistaken one. Our suggestion was, not that the nurse in charge of the country district had too little to do, but that as the nurse in charge of the town district has admittedly too much to do, the Society, with a balance of £74 to their credit, might be able to consider the idea of obtaining further help.

### OPPOSITION TO A NURSING GRANT.

AT a meeting of the Forden Board of Guardians Mr. J. Pryce Jones opposed a subscription of £2 2s. to the Montgomery Nursing Association, on the ground that one of the nurses attached to the Welshpool Institute—to which the guardians subscribe £5—refused to attend a pauper person until she got an order from the doctor. The conclusive reply to this objection was that the nurse, in refusing to take instructions from an individual guardian, was simply doing her duty. The case in question should, of course, have been mentioned to the committee of the Welshpool Institute, and no doubt the nurse would then have been instructed by the doctor to attend it. The Forden Guardians agreed, by a large majority, to subscribe to the Montgomery Institute, and the treasurer of the Welshpool Institute, who is a member of the Board, spoke in the warmest manner of the work done by the district nurses in that town.

### WARDSMAN AND NURSES.

The Inspector of the Irish Local Government Board, having held an inquiry at Mitchelstown Workhouse into charges preferred against a wardsman in the Union Hospital, announced at the termination that he should report that the accused was not "a fit person for the position of wardsman." Among the witnesses was the master of the workhouse, who said that the wardsman had acted in an insulting and disrespectful manner towards one of the nurses. His evidence was confirmed by the doctor, and two of the nurses were subsequently called. It was stated by one that the man swore at her in a most threatening manner, and called her by her surname. The other stated that he had used language most hurtful to her feelings, such as she could not repeat. The wardsman denied the charges, but the inspector considered them sufficiently proved to justify his report.

### CONFLICTING STATEMENTS AT WEST BROMWICH.

At a recent meeting of the West Bromwich guardians Alderman Williams stated that there was "scarcely a week went by without the resignation of a nurse being received." There must, he thought, be some cause for this, and he suggested that the matter should be inquired into by a committee. The master, however, affirmed that only two nurses had resigned during the last three months, and the deputy-clerk observed that there had only been two changes in the ordinary day staff of nurses during the past three years. In view of these conflicting statements the guardians decided not to take any action, but it is strange that Alderman Williams should have raised the question if only two changes have occurred in the course of three years.

### AN UNUSUAL CHARGE BY A POOR-LAW MEDICAL OFFICER.

The Scarborough Guardians at their last meeting had under consideration a bill for £10 sent in by Dr. Foley, medical officer of the workhouse, the amount being his charges at 5s. per head for the examination of 40 candidates for the position of nurse. The clerk to the Board informed the chairman that the claim was quite new in his experience, and after a brief discussion the matter was referred to the house committee. The house committee might do well to apply to the Local Government Board for instructions.

### PROGRESS AT BARROW INFIRMARY.

The Barrow-in-Furness guardians after long discussions on the standard of nursing in Poor-law institutions, assented to a proposal to appoint two trained nurses to fill vacancies caused in the workhouse infirmary. It has previously been the custom of the guardians to select for the position of junior nurse young women with very little experience of nursing, and they must be congratulated upon the step which, in spite of considerable opposition, they have undertaken. Of course, as Dr. Anderson pointed out at one of the meetings, it is an erroneous idea to suppose that nurses who commence in workhouse hospitals, such as at Barrow, can in time become highly-trained nurses, because that is impossible, unless theoretical as well as practical training is given. But a junior nurse, with "some training" in a hospital or workhouse infirmary, is preferable to one substantially without either, and the innovation at Barrow may pave the way in the future to more valuable reforms. The staff will in future consist of a superintendent nurse, two trained nurses, and a junior nurse. Hitherto, in the absence of the superintendent, there has been no trained nurse in the building, and we agree with Mr. Britton, the pioneer of the reform, that the Infirmary should never be without a trained nurse.

### MEDALS FOR PROBATIONERS.

Following the example of the board of the Huddersfield Infirmary and other institutions, the Bethnal Green guardians have decided to give two medals to be competed for by the probationers at their examinations, one silver medal to be awarded to the nurse who secures the highest number of marks at each examination, provided the same is at least 80 per cent. of the total number of marks allotted, and a bronze medal to the nurse who obtains the next highest number of marks provided she secures at least 75 per cent. of the marks allotted. The innovation is to be retrospective, and medals will be assigned to nurses trained at Bethnal Green Infirmary who have already satisfied these conditions.

### A HOME FOR PONTYPRIDD.

There has just been opened at Pontypridd a new home for the Queen's nurses working in the town, under the auspices of the District Nursing Association. The home is a gift from Miss Thomas, of Llwynmadoc, and was built at a cost of about £800. It has been furnished by means of subscriptions and gifts in kind, is commodious and comfortable, and will afford provision for an increased staff whenever the number of nurses can be augmented.

### VILLAGE NURSING IN FLINTSHIRE.

The Flint County Council are offering a number of scholarships for the training of women in village nursing. The amount of the scholarships covers all charges and expenses, and the training will be given at the hospital or institutions designated by the Council. When candidates have been trained they are to act as nurses in the county of Flint for not less than three years, and receive a salary of £38 per annum.

### SHORT ITEMS.

The Midwifery Scholarship for the past quarter at the General Lying-in Hospital, London, has been awarded to Nurse M. C. Smith for proficiency as a monthly nurse.—The Order of St. John of Jerusalem has been awarded to several matrons who had charge of the concentration camps during the South African War, including Miss M. F. Mackenzie, Miss Grace Mackenzie, and Miss Macgregor, all of whom were engaged at Dundee Royal Infirmary at the time the war broke out.

### The Nursing Outlook.

"From magnanimity, all fear above;

From nobler recompense, above applause,

Which owes to man's short outlook all its charm."

### THE SELECT COMMITTEE ON REGISTRATION.

It is strange to be once more sitting at Westminster and listening to the old quarrel between the two sections of the nursing world. Some 12 years ago, before a committee of the House of Lords, we had much the same evidence being brought forward, much the same persons as the audience, much the same spirit animating the opposing parties. And it is interesting to note that the Lords Committee of a dozen years ago decided by six votes to two against registration of nurses by the State; and that in the charter that was granted to the Royal British Nurses' Association state registration was refused, and it was suggested to the Association that their own system of voluntary registration was all that was necessary.

Have things now altered so far as to make us expect a change of opinion in the governing body? That remains to be seen; but meanwhile it is instructive to note the two lines of argument that are being brought forward by the witnesses now being examined at Westminster.

Those in favour of registration state that they want it as an "additional safeguard," and openly acknowledge that, in spite of a Government examination, they will have to go back to the hospital authorities for evidence of the character and training of the nurses who apply to go on the roll. And, as Mr. Sydney Holland said, a matron is not likely to give more than a very general character to a central body. It is one thing, when a doctor asks for a certain nurse for a young fellow with pneumonia, to say in confidence, "I have found her rather flighty, and prefer to send her only to women patients at present," and quite another thing to ruin a woman's prospects by telling a central authority that that woman's character is unsatisfactory. There are all sorts of practical difficulties to registration that become more and more evident every day the Committee sits. Miss Isla Stewart made a very damaging admission when she stated that the scheme of registration "would not include cottage nurses." If this be so, the scheme is only an additional safeguard for the rich and not for the poor; and the rich generally hire their nurses through the doctors and are plentifully protected already. An equally damaging admission on the other side came from Mr. Sydney Holland when he owned that he objected to registration as likely to make the nurse consider herself as the "colleague" of the doctor. In the whole of that part of his evidence he practically acknowledged that registration would probably raise the status of the nurse and make her regard her work as a profession. As the chairman, Mr. Tennant, acutely remarked, herein probably lay some of the medical animosity to registration of nurses. It is a difficult point. So long as the doctors expect a nurse to do their bidding first, to hold them above even the guide of conscience, probably it is well that the nurse's main training should be to blind obedience. In that amazing book, "The Confessions of a Physician," the pitiful nurse only turns her eyes aside when the incompetent physician is practically murdering a child by his clumsy efforts to perform tracheotomy. And if ever a nurse felt herself called on to criticise the medical world, and to publish the "Confessions of a Nurse," there might be much to shock the public. But, however much we desire to see nursing raised to a profession, we do not desire to see that consummation achieved by sacrificing the many to the few, by putting the excellent nurses working all over Great Britain at a disadvantage, by giving extra protection to the nurses of the big London hospitals, who are already so highly privileged.

There are members of the Committee who have evidently had some experience of district and country nurses, and are not inclined to allow the London nurse to have it all her own way. And Sir John Batty Tuke, who sits on the chairman's left hand, comes out with catching questions at times that show his practical knowledge of the subject. He has had much experience of male nurses and mental nurses, he is a member of the General Medical Council of Registration, and is the newly elected President of the Association of Asylum Workers. Other members of the Committee are Mr. Mount, Lord Morpeth, Mr. Pierpoint, Major Bagot, and Mr. Hobhouse. These must gain their knowledge as the inquiry proceeds. Theirs is the voting power, and therefore it behoves us to put before them all sides of the question and aid them in their decision. It is sincerely to be hoped that the personal quarrel will soon be got over and the unbiassed evidence be brought forward. Already such unsuitable suggestions as the training of probationers at 18 and a fee of five guineas for the examination have been made. Unless the evidence given is followed and more or less answered by other evidence we must not be surprised if the non-medical section of the Committee—and they are in the majority—get led astray. It is not enough for matrons or doctors or nurses to merely sign for or against registration. A little enthusiasm, a little sense of public duty, is necessary. The meetings of the Committee are open to all and are at present held on Tuesdays and Thursdays from 12 to 2 P.M. at the House of Commons, and those who cannot attend can follow the evidence in the nursing or medical papers or in the *Times*. But at least let us awake to the fact that the Committee is sitting, and that its deliberations must affect the future of the nursing world in one direction or another.

### Lectures upon the Nursing of Infectious Diseases.

By F. J. WOOLLACOTT, M.A., M.D., B.S.Oxon, D.P.H., Senior Assistant Medical Officer, Park Hospital, Metropolitan Asylums Board, Hither Green.

### LECTURE I.—INFECTION.

DISEASES which are transmissible from one person to another are said to be infectious. Such diseases are very numerous, and present great differences in character, but have the common peculiarity that directly or indirectly each case arises from a previous similar case. The infection may spread straight from the sick to the healthy or may be carried in articles of food or clothing or in other ways.

A knowledge of these facts is of the first importance, for if we understand the laws governing the spread of a disease we have made the first step towards checking its progress, and perhaps stamping it out altogether. It is advisable, therefore, to begin the study of infectious diseases by obtaining a clear understanding of the nature of infection, and then we shall be in a position to consider how it may be destroyed. Even up to recent years only vague ideas existed on these subjects, and the preventive measures adopted were often not much more effective than those used by savages to-day, who ascribe disease to the influence of evil spirits, and whose treatment consists of the free use of loud noises and bad smells. Light began to dawn when the microscope revealed the existence of a class of minute organisms known as microbes or bacteria, and step by step it has become more and more certain that it is to these organisms that infectious diseases owe their origin.

Without going into details, a brief account of these microbes will be serviceable. Owing to their extremely minute size they are only visible by the aid of the microscope, under which they appear as little dots, or short rods or spirals. They are of many varieties, each with its own peculiarities, and some of them are present everywhere on the earth's surface. Many of them have no ill effects on human beings, while others are able to produce disease and are therefore termed *pathogenic.* The latter group is the only one we need consider here. Several of its members have been closely studied and their relationship to disease clearly established. Diphtheria, for instance, has been shown to be due to the action of a certain special rod-shaped bacillus, whose presence can be demonstrated in the membrane that characterises the disease. The same is true of enteric fever, plague, and tuberculosis. In other cases, *e.g.* measles and small-pox, no characteristic microbe has been found; but, as time goes on, the number of such diseases is steadily diminishing, so that it appears probable that sooner or later an organism will be found in every instance. We may assume, therefore, that each infectious disease has its own corresponding microbe. Like all other living beings a micro-organism requires suitable food, moisture and warmth. If these be supplied it is able to multiply at an exceedingly rapid rate. In the tissues of the human body it finds all the necessary conditions, so that it may grow and thrive.

We are now in a position to understand the precise meaning of the word infection, and to realise that it implies the presence of disease-producing microbes. A patient suffering from such a disease as small-pox, for example, is the host of a large number of such microbes, some of which are constantly leaving his body. They may either attack a healthy person directly or may attach themselves to various articles in the sick-room or its surroundings. These articles then become infectious and serve as vehicles for spreading the microbes and with them the disease.

When a healthy person is exposed to infection one of two things may happen. Under favourable conditions, the resisting power of the body is such that the organisms are unable to develop their powers and soon disappear. On the other hand, they may find their new host but feebly protected against their attack. In that case, they thrive well at his expense, and in due course give rise to disease.

From exposure to infection to the first symptoms of illness, a certain interval always occurs, during which the microbes are increasing in numbers and overcoming the resistance of the body. This is known as the *incubation period.* It varies considerably in duration. Thus in scarlet fever and diphtheria it is less than a week, while in measles, and small-pox it is longer. After exposure to infection it is necessary to wait some time before all danger can be said to be over. This is known as the *quarantine period,* and corresponds to the maximum incubation period. Recovery from most of the infectious diseases confers protection for a time at least against further attacks of the same disease. This is known as *immunity,* and people so protected are said to be *immune.*

The agencies by which the germs of disease are spread are various and must now be noticed. Probably the commonest is direct intercourse between the sick and healthy, and the more close the intercourse the more sure is the disease to spread. Any custom that brings susceptible persons into close contact tends in this direction. School attendance furnishes an illustration of this. A child sickening with scarlet fever, for instance, has ample opportunities for infecting his companions, and it has been found that such diseases as scarlet fever and diphtheria are more prevalent among children when the schools are open than in holiday time. We now know that animals may be affected by some of the diseases that attack human beings and may therefore assist in their propagation. Thus cats may suffer from diphtheria, while rats and fleas are very active agents in the spread of plague. Infection may cling to any article which has come into contact with the sick. Especially is this true of bedding and clothing. The dress of the nurse may be at fault and instances are numerous of the transmission of disease by means of clothes, although the person wearing them may remain perfectly well.

Another and very important way by which infection may be spread is through the agency of food, especially milk and water. Many outbreaks have been traced to the former of these, the source of infection usually being some person employed in its collection or distribution. In other cases the disease has been traced back directly to the cow. Any article of food into the composition of which infected milk enters is a possible danger. Ice-creams are notorious offenders. The great importance of milk arises from the fact that it is able to supply any bacteria that may gain access to it with ample nourishment, so that they rapidly multiply. Unfortunately in many cases they cause no obvious change, so that their presence is not detected. Scarlet fever, diphtheria, enteric fever and tuberculosis may all result from infected milk.

The most important diseases spread by drinking water are cholera and enteric fever, and as a rule the mischief can be traced to contamination of the water with sewage. Such waters are able to communicate their infective properties to the oysters and other shell-fish growing in them; and of recent years it has been shown that many cases of enteric fever are due to the consumption of these articles of food.

The influence of season on the prevalence of infectious diseases is interesting. On the whole they seem to show a preference in this country for the last quarter of the year. Scarlet fever, diphtheria, and enteric fever, for instance, are at their lowest during the spring and early summer. A rise then occurs, which reaches its maximum towards the end of the year, after which a fall begins. The reason of this is not fully understood. Several causes are probably at work, such as the variations in temperature, the amount of rainfall, and the amount of moisture in the soil. It must not be forgotten, too, that in cold weather people stay more indoors and in closer association one with another, so that the spread of infection is facilitated.

### How to Become a Midwife: The Rules of the new Act Explained.

By E. L. C. APPEL, M.B., B.S., B.Sc.(Lond.).

Continued from page 208.

### III.—THE CERTIFIED MIDWIFE—HER RELATION AND DUTIES TO THE LOCAL SUPERVISING AUTHORITY.

CONTENTS.—The Local Supervising Authorities—Notification *before beginning* to Practise as a Midwife in her District —Notification of Cases Outside her District—Notification every Year, in January—Notification of Deaths, of Still-births, of Puerperal Fever and other Infectious Diseases, and of Medical Help sent for—Change of Address—Inspection of Midwife's Book, Appliances, etc.—Suspending a Midwife from Practice—Malpractice, etc.—Conviction and Prosecution of an Offence.

### *The Local Supervising Authorities.*

The Local Supervising Authorities are directed by law to exercise general supervision over certified midwives who practise as midwives in England or Wales. They are also entrusted with various other duties arising out of the Midwives Act. The certified midwife has certain duties to perform towards the Local Supervising Authority before she is allowed to begin to practise as a midwife in the district over which that Local Supervising Authority has control.

If, therefore, a midwife decides to practise in any part of England or Wales, she should first ascertain which of the local supervising authorities has charge of the district or area in which she resides or intends to practise as a midwife. The Local Supervising Authority in London is the *London County Council.*

The Local Supervising Authorities over the rest of England and Wales are the *County Councils* and the *County Borough Councils.*

But the local supervising authority may delegate any powers or duties conferred or imposed upon them by the Midwives Act to a *Committee* appointed by them and consisting either wholly or partly of members of the council. Women are eligible to serve on any such committee.

A county council may delegate any of the powers and duties conferred or imposed upon them by the Midwives Act to any *District Council* within the area of the county, and that District Council may appoint a *Committee* to carry out these powers and duties. This Committee may consist either wholly or partly of members of the District Council, and women are eligible to serve on the Committee.

Any one of the following five bodies may therefore be the Local Supervising Authority of a district or area :—

1. The London County Council.

2. A County Council.

3. A County Borough Council.

4. A District Council.

5. A Committee (on which women may serve) appointed by any of the four above-named councils.

The midwife must ascertain which of these bodies exercise the powers of the local supervising authority in the district in which she intends to carry on her practice.

### *Notification before Beginning to Practise as a Midwife in her District.*

When a midwife has decided in which district or area of England and Wales she intends to reside and carry on her practice as midwife, she should write for Midwife Form 12,[12] and after she has filled in and signed the form she should send it to the clerk of the body exercising the powers of the local supervising authority in her district.

*Form* 12 is as follows:—

MIDWIVES ACT, 1902, SECTION 10.

To the Local Supervising Authority of * the Administrative County of ..........................................................

*or the County Borough of..............................................

*or the Urban *or* Rural District of..................................

I, *A.B.*..........................................................................

*(*formerly*) ..................................................................(C.)

holding a certificate from the Central Midwives Board, No. ......, dated the..............of........................., 19..., hereby give you notice* (*a*) of my intention to practise as a midwife within your area during the year commencing January 1, 19....

*or (*b*) that on the..............day of...................in this year, I acted as a midwife at..........................., within your area.

.......................................... (Signed) *A. B.*

Residing at.......................................................and pursuing my calling at.........................................

Dated this.........day of..................., 19....

* Strike out the words not applicable.

In filling in and signing this form, the midwife must give:—

1. The number and date of the certificate which she received from the Central Midwives Board when she became certified.

NOTE.—This certificate is Form 11 in the case of a woman who became certified because at the passing of the Midwives Act she had been in *bonâ fide* practice as a midwife for at least one year.

The certificate is Form 10 in the case of a woman who became certified because she held a certificate in midwifery from an institution or examining body approved by the Midwives Board.

The certificate will be Form 2 for a woman who becomes certified after March 31st, 1905.

2. Her Christian name and surname in full, and if she has married since the certificate was granted to her, she must give also the name under which it was granted to her.

3. Her usual place of residence, and if she carries on her practice elsewhere she must give also the address where she practises.

The following is an example of the way in which Form 12 would be filled in and signed by a midwife whom we will imagine to be practising as a midwife in the rural district of Uckfield, whose certificate number and date we will imagine to be 15 and August 5, 1903; whose name and surname in full we will imagine to be *Mary Anna Smith* now, but when the certificate was granted to her *Mary Anna Thomas*; and whose place of residence where she also carries on her practice we will imagine to be 18 High Road, Duddleswell:—

MIDWIVES ACT 1902, SECTION 10.

To the Local Supervising Authority of the Rural District of Uckfield.

I, Mary Anna Smith

(*formerly) Mary Anna Thomas .............................. (C.), holding a certificate from the Central Midwives Board,

[12] All Midwife Forms (price 1d. each, postage extra) can be obtained from The Scientific Press, Ltd., 28 and 29 Southampton Street, Strand, London, W.C.

No. 15, dated the fifth of August, 1903, hereby give you notice of my intention to practise as a midwife within your area during the year commencing 1st January, 1904.

(Signed) Mary Anna Smith.

Residing at 18 High Road, Duddleswell, and pursuing my calling at 18 High Road, Duddleswell.

Dated this 8th day of January, 1904.

* Strike out the words not applicable.

[These articles are published in book form by the Scientific Press, Ltd., 28 and 29 Southampton Street, Strand, W.C., at 1s., and 1s. 1d. post free. All midwife forms, price 1d. each, postage ½d. extra, can also be obtained from the Scientific Press.]

(*To be concluded.*)

### The Registration of Nurses.

The Select Committee of the House of Commons appointed to consider the expediency of providing for the Registration of Nurses, met on Thursday the 14th and again on Tuesday the 19th instant.

On Thursday Sir Charles Burt, on behalf of the Central Hospital Council for London, presented a copy of a resolution passed by that body, and signed by 313 persons, opposing the principle of State Registration.

### Mr. Sydney Holland Examined.

The Hon. Sydney Holland then gave evidence, claiming, by virtue of great experience in hospital and nursing matters, to have a right to speak with authority on the subject of registration. It would, he maintained, be hardly likely that persons whose lives had been devoted to nursing, and the interests of nurses, would oppose registration if it appeared to them likely to improve nursing in any way. Referring to two previous discussions of the subject, Mr. Holland said that the House of Lords, in their 1902 report, voted by a majority of six to one against it, while the fact that the Privy Council had refused it to the Royal British Nurses' Association, when granting them a Charter, was significant that, in their opinion, registration should be voluntary.

The Chairman having remarked that the Committee, while attaching great importance to the authorities quoted, were not prepared to accept their decisions as the guiding principle, Mr. Holland instanced the manifesto against registration, which he believed had been signed by the matrons of all but three of the London hospitals, as well as by many other persons, the weight of whose opinion could not be denied. While cordially acknowledging the sincere desire, on the part of the promoters of registration, to act in a manner which they believed would advance the interests of nurses, he asked the Committee to satisfy themselves as to the experience of these people, and above all not to imagine that the opinion of matrons generally was represented by a small self-elected body, the name of which was apt to convey a false impression. In reply to a question, the witness said there was no council really representative of the matrons, but the one alluded to was first in the field. Continuing, he said that registration would be no safeguard to the doctor that he would get a good nurse; while it would lead to the idea among nurses that by belonging to a profession they were elevated to the rank of a sort of pseudo-scientific assistant of the doctor. Neither would it safeguard the public, since only the rich would be benefited, this being the very class already in a position to secure good nurses by full inquiry, the only possible safeguard. Registration, he maintained, would tend to stop inquiry. The State would be taking upon itself the responsibility of a continuous guarantee, and the fact that a woman had passed an examination would, 10 years later, be no proof that she was abreast with modern methods of treatment. A nurse, moreover, might drop out of sight for years, but on re-entering the field of work she would still retain the guarantee given by the State, while in knowledge, and possibly character, she might have become hopelessly incompetent. He could not agree that the three years' training insured continuous capability, as had been asserted. With regard to the certificate of moral fitness, which it was suggested must be given by the hospital where the nurse received her training, Mr. Holland said that such certificates would necessarily be of a very general character, and would not even be a guarantee of technical skill. Deterioration of character was a difficulty with which all connected with nursing were familiar. From personal experience he was convinced that examination was no guarantee of fitness to nurse; indeed, in some instances the least brilliant in examination proved the best nurse.

What, he asked, was it proposed to register? A very high standard would exclude a number of excellent women, while a low one would be useless as a safeguard. He thought no one seriously proposed a graduated standard. A "uniform standard" was a very misleading expression, since three years in one hospital by no means corresponded with the same term in another; 20 years in some would be no training at all. It was a great mistake to fix upon a term of years, and it was absurd to keep a woman out of responsible work merely because she had not served her time. Registration would, in his opinion, tend to "grind" and undue haste on the part of the nurses, and to a dead level on that of the training schools. A slur, moreover, would be cast on the many women who were doing excellent work throughout the country, but who were not qualified to go on the register.

Dealing with the question of supply and demand, Mr. Holland said the recent developments—*e.g.* in Army and Poor-law nursing—had made great demands on the number of available women offering for the work of nursing; they did not exist in sufficient numbers to meet the requirements of registration. The high fee suggested would tend to keep out many nurses; few people realised how poor nurses were. At the request of the chairman he gave full details of the scale of pay, pensions, etc., in force at the London Hospital. He could not understand how anyone could suggest that a girl should begin her training at 18, going straight from school to the sights and sounds of a hospital, with no interval for home life. Probationers were not accepted at the London under 23.

The Chairman said that did not affect the main question.

### Questions by the Committee.

The evidence being concluded, questions were put, the Chairman thanking Mr. Holland for the information he had given. The Committee recognised the debt of gratitude which the public owed to those who gave their time to the hospitals. The witness, of course, would admit that there were people who had done this, but who held opinions differing fundamentally from his own.

Mr. Holland asked why the opportunities of voluntary registration afforded by the Royal British Nurses' Association and by Sir Henry Burdett had not been taken advantage of to a fuller extent; he admitted, however, that State registration might have superior attractions for nurses. If, as the chairman suggested, the idea that the registered nurse would think herself a pseudo-scientific assistant to the doctor, accounted for the opposition of many of the medical profession to registration, he considered that a very good reason. There was a great danger to the public in a nurse thinking herself equal to a doctor. Being asked if he were satisfied with the present position of nurses, Mr. Holland said he was, but that did not mean that he did not wish for any improvements. If people failed to arrive at the desired information about a nurse, it must be that they went the wrong way to work. A hospital, he held, was legally responsible for the actions of the nurses it supplied, and was in a very different position to the State in having special knowledge about the women trained by it. Although matrons changed, as the chairman pointed out, yet by keeping a careful record of every nurse trained, with all available particulars of subsequent history, each hospital ought to be in a position to give full information when applied to. State registration, he urged, would constitute a false hall mark; the real one could only be given by the training school. A revision, say every ten years, would be impracticable, with a roll of thousands of nurses.

Some difficulty seemed to be felt by the Committee in accepting Mr. Holland's view of the difference between the certificate of the London Hospital and one given by the State; one member suggested that the proposed certificate might make it clear that it only guaranteed that a certain examination had been passed. The chairman asked whether it was not possible, under existing circumstances, for a nurse who had become disabled in some way to go about with her hospital certificate, to which Mr. Holland replied that this was so, and that with an additional certificate from the State such a woman would be a worse danger still. He thought, however, that the complaints were exaggerated, and where such cases were found to exist, the remedy was to encourage inquiry.

With regard to the effect of registration on numbers, he held that it would make no difference at all. Educated women were increasingly difficult to obtain as nurses, and he scouted the idea that they would be attracted by the prospect of an improved "status." There was a status already, and they knew that the best posts were open to them.

There was considerable discussion on the question of the removal of unsatisfactory women from the proposed register, and on the suggestion that the Council, equally with the London Hospital, might exercise its discretion, Mr. Holland questioned the possibility, in the face of overwhelming numbers. The chairman said that the cases were parallel, and Sir John Tuke said that the names might be dropped out of the list.

Mr. Holland did not agree that any analogy existed between the case of nurses and that of midwives, who were trained for one particular duty; he had been strongly in favour of registration for the latter.

The Chairman asked to what extent partially trained nurses were sent out—as stated in the Lords' Report of 1902—from the London Hospital to private cases, in reply to which Miss Lückes, who was present, explained that it was only done at that time at the request of the medical staff, to meet a particular need, and never to any great extent.

After some further particulars as to the private staff at that hospital, in the course of which Mr. Holland said the fees figured in the accounts as some £4,000 gross receipts, the Chairman said the subjects dealt with were of so great importance, and the matter so interesting, that he would ask the witness to appear again. The Committee then adjourned.

Continuing his evidence on Tuesday, Mr. Holland said that it would be impossible to make an examination in nursing which an intelligent woman could not pass in six months: the limit of knowledge was so small, and nursing examinations so childishly simple. He did not say there was no advantage in registration, but that it was a very small one—*i.e.* that of enabling a doctor to telegraph for a registered nurse; and this did not insure his getting a good one. Referring to the decision in the Court of Appeal, given since Thursday's sitting, he thought that the opinion of the Attorney-General as to the liability of a hospital or association supplying a nurse should be obtained. Replying to questions, he said that while it was a commonly held opinion that three years' hospital training unfitted a woman for district work, a really good woman would do any kind of work. He did not think anyone would suggest that there should be two classes of registered nurses. Asked whether he would do away with the minimum training for Army nurses, Mr. Holland said he thought not; indeed, he had been one of those approving of the recent fixing of a two years' training or one year's service. But it was not the number of years spent in a hospital that made a good nurse. The number of hospitals from which these nurses were taken was limited. He was understood to add that Army nursing was highly skilled.

### Evidence of Dr. Norman Moore.

Dr. Norman Moore opposed State registration on the ground that it would tend, by setting up a fixed standard of attainment, to impair the progress in nursing generally of the last few years. He saw no danger in a lack of uniformity in training. He had not personally heard any complaints that untrained women were competing with the fully-trained, and as far as his experience went, he considered the position of the best nurses at the present day a very good one in every way. Registration, in his view, would lead to the establishment of an imperfectly-trained order of medical practitioners, and complaints, which he had often heard, that people employed nurses rather than doctors, would be exaggerated. The responsibility in the treatment of disease rested with medical men, and they would be extremely wrong, under any circumstances to part with it. The Chairman pointed out that the object of the committee was to secure the improvement of the nurse in her capacity of carrying out the doctor's orders; there was no suggestion that she should give orders herself.

Dr. Moore dwelt on the difficulty a large body—*e.g.* the General Medical Council—experienced in sifting evidence on matters verging on the criminal; he thought that the proposed Central Board would find that, apart from crime, nothing could be done to remove undesirable names from the register. Under existing circumstances, it was the province of the medical man to employ only such nurses as he was convinced were competent, and to avoid employing, or allowing to be employed, those whom he knew to be incompetent. Enough stress had not been laid on the effect of the *esprit de corps* of a good training school in keeping nurses up to the mark. Properly conducted examinations would be not only very expensive, but would tend to special reading on the part of the nurses, and book knowledge alone was of little value. The door should be kept as widely open as possible, and nothing should be done—by high examination fees or by any other obstruction—to discourage women in any rank of life from becoming nurses. Women of the domestic servant class, for example, frequently made admirable nurses. With regard to a three years' minimum, this was unnecessary in the interests of the public, and in the present state of things it would be unwise to insist upon it. Some of the work in country districts could be usefully done by women with six months' training. He did not think that the question of a nurse telegraphed for by a doctor on a Yorkshire Moor, or in the Riviera, an emergency suggested by Dr. Hutchinson, need affect the question. He hoped that an isolated nurse would always send for the doctor.

The Select Committee will not meet after July 26.

### Nursing a Patient with a Protracted Illness.

### EXAMINATION QUESTIONS FOR NURSES.

The question was as follows:—"Give an account of what you would consider the most satisfactory room in which to nurse a patient with a protracted illness, and how—speaking broadly—you would like it to be furnished, ordinary maladies, not those of an infectious nature, being under consideration."

### First Prize.

I should choose the position of the room in the house to be not higher than the third floor, second floor preferable, for the sake of getting the patient out of doors easily when the convalescent stage is reached, and also for the sake of the legs that must fetch and carry during the illness.

I should like two large windows in it (with sun-blinds outside), facing south or south-west, and with a quiet outlook.

Ceiling high and walls papered with sanitary paper of some cool art shade or of inoffensive pattern. A few pictures of pleasant subjects and of such a size and so hung that their position could occasionally be changed should the patient desire it.

Two beds of moderate size placed side by side, the distance apart to be regulated as required either for the use of the nurse or for changing the patient, placing him on a water-bed, or airing the bedding. I should place the heads of the beds against the wall at right angles to the windows, feet out into the room, so that the patient may either face the light or turn his back upon it as he pleases.

The fireplace in the wall facing feet of patient.

Door (with screen) opposite the window furthest from patient's bed so that the feet shall be in no draught though a through breeze may be allowed to blow through the room if required.

Ordinary bedroom furniture, washing-stand, wardrobe, bookshelf, etc. Two small easily moved tables, one or two comfortable chairs for visitors.

The floor stained, but plenty of rugs about which can be easily lifted and carried from the room for cleansing purposes without disturbing the patient by sweeping in the room.

A couch or sofa, light enough to be drawn up to the window or to the fire for the use either of the patient at the convalescent stage or for a nurse at night.

A bed table, and if such luxuries are allowed, an electric bell and shaded electric light, so placed and arranged that the patient can use them at will from his bed.

Sandy.

### Second Prize.

An invalid's room should be bright and airy with an open fireplace and well ventilated. It should receive plenty of light and sunshine and be as far as possible away from noises. The higher up a room is the better it is for fresh air and ventilation (everything else being equal). There should be two large windows, or, if very large, one might be sufficient.

If possible an adjoining room should be set apart to contain utensils, medicines, dressings, etc., in fact everything that would be at all disagreeable to the patient.

Here also any poultices, dressings, etc., required may be prepared.

This room should contain, apart from the utensils, etc., a chest or wardrobe for linen, a table, and a cupboard. A gas ring would be useful.

If such a room cannot be obtained, a corner of the landing outside or of the sick-room could be screened off. The latter is not, however, desirable.

The walls and ceiling of the sick-room would be best painted—a quiet restful colour in some neutral tint—they can then be frequently washed.

If room must be papered a plain paper is desirable, as a pattern is often irritating to an adult patient. For a child, unless complete rest for the brain is necessary, a paper on which nursery rhymes are pictured often helps to amuse. Two or three good pictures might be allowed, restful landscapes or faces, on no account war pictures or the like.

The floor might be stained and two or three art rugs will give an air of colour and comfort to the room, and can be taken up and shaken.

If the floor is not suitable for staining, linoleum may be used, as it can be easily washed. The art rugs would still be required.

If space permit there should be two single spring-beds, so that the patient can be lifted from one to the other to have bed made and aired. If there is not room for two beds the lounge must answer the purpose of one.

The bed for the time in use should be placed away from the door, should be accessible from all sides, and should be where the sun from the window will fall pleasantly, though not too strongly upon it. There should be no bed hangings.

A bedside table and a washstand will be required; for the latter one of the small iron ones that can easily be brought to the bedside is most convenient.

Two ordinary chairs, one easy chair or lounge, and a screen will be all the extra furniture required.

The room may be made to look bright and cheerful by the addition of potted plants and cut flowers; these latter should not have a strong scent, and both plants and flowers must be removed from the room during the night.

Long curtains of some soft washing material give an air of finish and cleanliness to the room.

A large number of ornaments and bric-à-brac must not be permitted in a sick-room, but if the patient is allowed to read, a small book-case or shelf containing suitable books might be allowed.

"Guernsey."

### Merits and Defects of Competitive Papers.

The work sent in this month gives evidence, on the whole, of careful thought, but there is one general fault. The candidates seem to forget that it is very rarely indeed that one knows beforehand when serious illness is at hand, so that it would be possible to stain floors and paint walls in desirable colours. It is true that the question asks for information as to your views on the most satisfactory room in which to nurse a long case, but it is necessary for nurses to read between the lines, and see that they must chiefly attempt to make the best of what they have, and this but seldom includes such delightful and spacious rooms as "Aubrey" and one or two other writers very sensibly desire.

I should therefore have preferred answers that dealt a little more broadly with the question, stating the need of a quiet but sunny room, linoleum or polished floors if possible, if not, clean boards with rugs; and it would have been better if more stress had been laid upon the position of the bed or beds, with regard to avoidance of draught and superfluity of light on the patient's eyes.

"Sandy" wins the first prize, because she mentions the desirability of two beds in the room when possible, and also because she makes provision for the patient turning from the light if he wishes to do so.

In the event of its being imperative that the bed should be either head or feet directly to the window, it is unquestionably better for the head to turn its back that way; light continually in the eyes is very trying, and makes reading almost impossible.

Both "Sandy" and "Guernsey" also see the need of placing the bed carefully so as to avoid draughts.

### Honourable Mention.

This is gained by "Newo," "Quetta," "Aubrey," and "A Beginner."

There will be no further questions until October.

The Examiner.

### Tending the Victims of the "General Slocum" Disaster.

By M. ARD MACKENZIE, Superintendent of the Brooklyn Hospital Training School.

If the disaster to the *General Slocum* had not occurred it would scarcely have been believed that it could take place so near the city and yet the ruin be so complete in so short a time. Of the 1,500 excursionists who left their homes on that fateful Wednesday morning nearly 1,000 were lost, and none returned unbereaved. The tragedy was the more tragic because so many of the victims were burned beyond recognition, and so few were left to receive the skilled and tender care of the doctors and the trained nurses who were so anxious, as far as human help could, to alleviate their sufferings.

When the burning vessel was descried word was instantly sent to the hospitals near the scene of the catastrophe, and preparations were made for every emergency that might arise. The Lebanon Hospital opened its dispensary, and a band of skilled nurses were stationed there to assist the surgeons with dressing of the wounds, burns, and other injuries. Eight nurses were despatched in the ambulance to the scene of the disaster itself, well equipped with all emergency supplies—oil, stimulants, sterile dressings, bandages, hypodermics, and instruments—and in a very short time were at work on North Brother's Island, where the scene was one never to be forgotten. There on the beach the doctors and nurses from the Bellevue, the Lebanon, the New York, Lincoln, Harlem, and North Brother's Island Hospitals knelt and dressed the burns and wounds and comforted as best they could, the heart sufferings of the afflicted, as body after body was washed or carried ashore. For hours they worked unceasingly, separating the dead from the living. The living they hurriedly sent on to the hospitals, the dead they laid aside as tenderly as possible, with their faces covered, to await identification. The Lebanon Hospital cared for from 150 to 200 cases, most of whom were able to return to their homes, after their wounds had been seen to.

The doctors, nurses, and other rescuers worked magnificently. Self was forgotten, as they gave themselves up to the work of rescue, wading in to seize bodies and to bring them to shore, hoping still to find the spark there, and fanning that spark so carefully lest after all it should escape them.

And so the night wore on, and the sea gave up its dead more and more reluctantly. It was arranged that the bodies should be conveyed to the improvised morgue on the Charities and Corrections Pier. Here for two days and two nights the march of the searchers for their dead was kept up. Tramp, tramp, until the heart of the listeners too grew sick. The awful silence of the stricken ones was heart desolating; no loud lamentations, no weeping, just that quiet, dry-eyed grief that stifles. One man came who had been tramping for hours; his search not complete, because, though he had found the bodies of his six children, he still sought his wife.

Later, in the tenements in the parish of St. Mark's, there was hardly a room without its casket, and in some homes, six, seven—nay, in one, nine—coffins were counted.

The whole city was stirred to its depths, and money poured in for the relief of the survivors, and many settlement workers, church visitors, school inspectors, and nurses volunteered their services. A relief committee was hastily organised. The work of relief was carried on most generously and, withal, most wisely. The chief assistance given was in defraying funeral expenses. Now the Committee is thinking out a wise and equitable plan of using the 90,000 dols. which remain, so that no needy one will be overlooked, and no harm will be done by needless giving of alms.

The clubwomen of New York have offered to pay the salary of a deaconess to work among the people of the parish, as that seems to be what is needed most, nearly all the workers of the church having been carried away. Out of 48 teachers, only six returned, and a like proportion of the 125 members of the Ladies' Aid Association.

Now, when we look back, we see that good will come out of this great calamity. There will be a more rigid inspection of excursion boats than heretofore, and some decided effort will be made to regulate undertakers' charges, so that there will not be a repetition of the exorbitant and wholly unreasonable demands made by the undertakers at this time of widespread affliction.

### Central Midwives Board.

### THE NUMBER OF EXAMINATIONS.

An adjourned meeting of the central Midwives Board was held at the offices on Thursday last week. There were present:—Dr. Champneys (chairman), Dr. Cullingworth, Mr. Ward Cousins, Miss Oldham, Miss R. Paget, Dr. Sinclair, and Mr. Parker Young.

The consideration of the examination scheme drafted by Dr. Cullingworth, before the Board at the previous meeting, was continued. Several clauses were postponed until the whole scheme can come up again *en bloc* for final approval.

The question of the number of examinations to be held in a year was then raised, and its reconsideration was moved by Dr. Cullingworth, seconded by Miss Paget, and carried. Dr. Sinclair moved a resolution that the examinations should not take place more often than twice a year; he objected to the policy of "providing facilities for failures" to come up again, but no seconder was forthcoming. Dr. Cullingworth said that since the Board considered this matter at the last meeting he had received important representations from institutions engaged in the training of midwives showing that if examinations were held only twice a year very great inconvenience would be felt by the schools, involving difficulties in management which would militate against the efficiency of the training. He considered the reasons given so conclusive that he had altered his opinion. After some further discussion, in the course of which Dr. Champneys expressed the opinion that the training would be better if pupils were turned out more frequently in small batches, Dr. Sinclair begged the Board to think of "highly-trained nurses," not of "charwomen," and Mr. Parker Young put in a word for those women who had saved up what for them would be a large sum of money to pay for their training, and who deserved some consideration. A resolution in favour of examinations being held four times a year simultaneously in London and the provincial centres was moved by Dr. Cullingworth, seconded by Miss Paget, and carried, Dr. Champneys and Miss Oldham also voting for it. A strong expression of opinion had come from the Queen Victoria's Jubilee Institute, which was read by Miss R. Paget, who represents the Institute on the Board.

It was agreed to defer the final consideration of the scheme until October.

The following institutions for the training of midwives under section C of the rules were approved:—The Belfast Union Workhouse, the County and City of Cork Lying-in Hospital.

A number of applications from teachers under Rule C.I. (3) and from midwives for the purpose of signing Forms III. and IV. under Rule C.I. (2) were postponed, it being agreed that copies of the schedules giving the qualifications of applicants should be sent round to each member of the Board, a course to be followed in future with all these applications. Miss Paget gave notice of a resolution she wished to bring up at the next meeting, that midwives should be required to renew their license annually.

Mr. Ward Cousins asked leave to postpone a motion limiting appointment as inspectors under the Board to members of the medical profession. Forms of notification of the death of a mother or child, and of the birth of a stillborn child under Section E, Rule 18, were approved, and ordered to be sent to the Privy Council for sanction.

Dr. Cullingworth stated that the Somerset County Council, one of the few Councils which had delegated its supervising powers, had, in consequence of the views expressed by the Board, rescinded its resolution, and agreed in future to retain them in its own hands.

### Everybody's Opinion.

[Correspondence on all subjects is invited, but we cannot in any way be responsible for the opinions expressed by our correspondents. No communication can be entertained if the name and address of the correspondent are not given as a guarantee of good faith, but not necessarily for publication. All correspondents should write on one side of the paper only.]

### THE TRAINING OF PROBATIONERS AT WARRINGTON INFIRMARY.

C. E. RICHMOND, F.R.C.S., hon. secretary of the medical committee, writes from the Infirmary and Dispensary, Warrington:—In your issue of July 16 you had a paragraph "The Training of Probationers at the Warrington Infirmary." This is misleading, as the workhouse hospital should have been indicated and not this institution, which is the Warrington Infirmary. Of course the statements under your heading are totally inaccurate as regards this institution. A correction in this matter is necessary, as it might convey an utterly false impression to any probationers applying for training at this institution.

[We print Mr. Richmond's letter, but we cannot conceive any probationer confounding the Infirmary and Dispensary at Warrington with the Poor-law Infirmary, which was unmistakably the subject of our note. "The Warrington Infirmary" was not mentioned.—ED. HOSPITAL.]

### A QUESTION OF SALARY.

"EVELYN L. C. EDEN" writes from Kingston, Somersetshire: In your article on the "Dangers of an Irresponsible Charity" you say "it is perfectly easy to get Queen's nurses if a decent salary is paid," and you imply that no nurse who has had less than a Queen's nurse's training should be employed on responsible district work. May I be allowed to give my experience in this matter? It is, of course, merely a question of funds. No one, except a certain number of ladies who have had no professional training, and some old-fashioned doctor, believes that an untrained nurse is better than a fully-trained one. A few years ago, coming fresh from hospital life, I held out tooth and nail for a graduate nurse; but I have lived to discover that in the country it is a practical impossibility in most parishes. We live in an exceptionally wealthy village, and, thanks to several large subscriptions and much labour on the part of the secretary, we can afford to pay our nurse from £75 to £80 salary. But I have reluctantly been convinced that there are few surrounding parishes that could do the same. What is therefore to be done? I know parishes that are struggling vainly to raise even £60 for a nurse. The Queen's nurse being unobtainable, is there to be one of inferior status or none at all? I am speaking of country requirements only. It must be remembered that, though, for a given area, cash is rare in the country, yet a more responsible kind of nurse is required, as the doctors are not within easy walk. It is therefore a difficult question. The nurses employed by Lady Margaret Bickersteth appear to be below any justifiable standard, and such need not be considered. It would undoubtedly be best if the country could be nursed from properly distributed centres instead of parochially. There would be much saving of money and time. In the meanwhile the Queen's Institute seems to have found some solution to the difficulty by encouraging the formation of county nursing associations. These employ women trained especially for district and maternity work for at least a year, and appoint a superintendent who supervises their work and continues to instruct them. At present there seems no other way out of the wood. May I, in conclusion, point out that it is not only partially-trained nurses who burn their patients! I have heard in the last few months of a nurse in a first-class London surgical home, and of a lady doctor, doing the same.

### RED TAPE IN THE WARDS.

"BET" writes: The article under the heading of "Red Tape in the Wards" was, I feel certain, written by someone who does not in the least understand the nursing outlook. I should not be surprised if it were written by that sarcastic visitor who noticed the immaculate gowns of the nurses and the spotlessness of the wards. I was trained in one of the large London hospitals, and yet I was never led to think of myself first. A nurse who is worthy of being so called will deem it a duty to look as immaculate as possible; it is expected of her. Otherwise, why is a uniform provided? My opinion is that the patients appreciate it as much, or more than any one. Again, the nurse must keep her ward up to the highest mark of cleanliness, but in doing this she need not neglect her patient. In the hospital where I was trained such a proceeding would not have been permitted. The remark about cream being skimmed from off the patients' milk for sister's tea is utter nonsense. I think I am not far wrong when I say that in all or in the marjority of our large hospitals the milk is sterilised before it is sent to the wards; in fact, as a rule, as soon as it is brought into the building. Such being the case, it would be impossible to skim cream from it. In my large hospital the "sisters" and "nurses" were allowed non-sterilised milk, thereby getting what cream was due to them. The detail from "another hospital," where the patient complained of cold feet, is one I always found easy to remedy by putting an old light blanket (we kept them for this purpose) next to the patient, so that in making the bed I could wrap it round the patient's legs and feet and thus avoid cold and draught. It is not because the beds look tidier that surgical beds have the blankets turned back, but to relieve unnecessary jarring to the patient, such as would be caused by frequently hoisting up the mattress to tuck the blankets under. Of course a nurse likes to keep her ward and beds tidy; if it were otherwise I do not hesitate to say that the sarcastic visitor would be the first to complain that the "nurse must indeed be without method or else be lazy." When I gave cod-liver oil I gave it in a medicine glass (I always had four or five more glasses than I had patients), but I carried a basin of common salt and a teaspoon on my tray with the glasses, and after the patient had swallowed the oil I put a little into the glass to absorb any that was left clinging to it, and then I washed it under a tap of running hot water and no trace of oil was left. We were supplied with small bottle-brushes for cleaning the spouts of our feeding-cups; and, from personal experience, I can say that milk or beef-tea drunk from one tasted as nice as it would if drunk from a Dresden china teacup or a cut-glass tumbler. Our patients were expected to bring a tooth-brush, and there were new tooth-brushes to be got at the clerk's office for patients who could not afford to buy them. There was no difficulty in giving patients warm water, as there was a tumbler for each patient's use, so that when warm water was given for washing the tumblers were also filled. If patients were not able to clean their own teeth the nurses cleaned them for them, or, if this was impossible, as it was sometimes with a delirious patient, we gave the mouth and gums a thorough swabbing with some antiseptic lotion diluted with hot water. I was never told to "hurry up" when making a bed, though I have often found it necessary to hurry, but always reserved the hurrying up until I came to the beds of patients who were able to sit on a chair. I am doing private nursing now, and have been doing so for two years and six months, but I have not yet been reported to my doctors because I have not been able to clean my patients' teeth, nor because I have not made their beds comfortably. But upon several occasions I have been complimented on the capable way I have made beds without tiring the patient, and I have nursed several medical men having been recommended by one to another. The West End patient must indeed have been worldly to cry because she looked like a Dutch doll. Did she consider her appearance before her life, and expect her nurse to do the same? I think the nurse by doing the two plaits added materially to her patient's comfort and gave her a chance of being less delirious than she would have been if the nurse had tried to arrange a smart West End coiffure; indeed, no nurse would think of such a proceeding. I speak, I am sure, for all trained nurses, because all our large hospitals train their nurses to be nurses in every sense of the word. I am also sure that the cap mentioned to be worn by those whom it might fit will go a-begging.

### Appointments.

[No charge is made for announcements under this head, and we are always glad to receive, and publish, appointments. The information, to insure accuracy, should be sent from the nurses themselves, and we cannot undertake to correct official announcements which may happen to be inaccurate. It is essential that in all cases the school of training should be given.]

Bury Union Hospital, Lancaster.—Miss A. E. Storey has been appointed superintendent nurse. She was trained at the Royal Infirmary, Newcastle, and in midwifery at the Rotunda Hospital, Dublin; has been charge-nurse at the Park Fever Hospital, London, and has since been engaged in private and district nursing. She holds the L.O.S. certificate.

Fulham Infirmary.—Miss Annie Little has been appointed ward-sister. She was trained at St. George's Infirmary, Fulham Road, and has since been charge-nurse at the City of London Infirmary, Bow; charge-nurse at Bethnal Green Infirmary; charge-nurse at Islington Infirmary; and maternity nurse at Westminster Union.

Isolation Hospital, Penarth.—Miss Sarah Parker has been appointed matron. She was trained at the Royal Infirmary, Manchester, where she has been staff-nurse. She has since been sister at the Convalescent Hospital, Cheadle, Cheshire; sister at the Royal Infirmary, Manchester, and sister and deputy-matron at Croydon Borough Hospital.

Northampton General Hospital.—Miss Amelia Evans and Miss Edith Grocott have been appointed sisters. They were both trained at the Liverpool Royal Infirmary.

Queen Victoria Royal Infirmary, Preston.—Miss Florence Eggins has been appointed night sister. She was trained at Worcester General Infirmary, where she has since been sister for two years.

### Presentations.

Bury Union Workhouse.—Miss M. E. Ball, who has resigned her post of superintendent nurse after 4½ years' service at the Bury Union Workhouse, has been presented by the nursing staff and a number of the officers with a handsome travelling-bag. A silver-backed brush and comb were given by the medical officer, Dr. Mellor.

### Wants and Workers.

The Secretary of the Chelsea Branch of the Association for Helping the Physically and Mentally Defective, 48 Wetherby Mansions, S.W., would be grateful for the gift of a secondhand self-propelling chair, for the use of crippled cases in this district, or a small sum could be paid.

### TRAVEL NOTES AND QUERIES.

By our Travel Correspondent.

In the Bay of St. Malo (Chislehurst). The numbers you want in 1899 will come to 3d. with postage. All the following addresses would, I think, be suitable for you. Write on a return foreign letter card and ask for terms and particulars: Mme. Pallot, Maison Mathias, St. Servan; Miss Humphrey, 20 Place Constantine; Mlle. Esnon, Les Myrtes. At St. Enogat, close to Dinan, the terms are low, and for two people, for a period of two or three weeks, I think they would meet you in terms. There is nothing suitable in St. Malo itself, and it is very much shut in and stuffy in the old town. St. Malo, St. Servan, Dinan, and St. Enogat are all near together and are connected by a continual service of small steamers across the bay. The two first are separated only by a dock, which is crossed in a kind of Noah's Ark on wheels for the sum of one half-penny. I do not know to what convent you allude, I mention so many. Where was it? Send the money for the numbers you want to the manager.

Normandy Coast (Caen). I fear there is nothing at your terms on the Norman coast. Farmers in France very rarely take lodgers. Dieppe is very dear and the country not interesting. Why not Brittany? it is much more generally picturesque. See answer to Chislehurst. Would you like Avranches? not sea exactly, but to some people a very charming place. If so, write to Pension Née, 54 Rue de la Constitution. I cannot give you any address by the sea in Normandy at your terms, but I could in Brittany, so let me hear again if that suits you.

A Fortnight in Paris (Salu Infirmorum). See answer also to "Doreen."—I know of nothing quite so cheap as you want to find. I do not think it exists. How can it, indeed, if the proprietor is to make even a modest profit in such a place as Paris? The following addresses approach your terms, and I know the houses to be perfectly respectable, as they are recommended by "The Teachers' Guild":—Mme. Morand, 13 Rue Washington; Mme. Garonne, 60 Rue des Ecoles; Pension Delarue, 7 Rue d'Assas.

A Few Days in Paris (Doreen). See answer to "Salu Infirmorum."—It is but a vague term to use when you say you want cheap accommodation, because what you call cheap I might call dear, and *vice versa*. If you can pay 7 frs. per day write to Mme. Ledoux, 20 Rue Clairant, Avenue de Clichy, Batignolles. For 8 frs. per day, you could go to the Hôtel Britannique, 20 Avenue Victoria, and if you are in Paris only a few days an hotel is preferable to a private house. The guide that would suit you is "Pearson's Gossipy Guide to Paris," 1s. Write to the publishers, C. A. Pearson, Limited, Henrietta Street, W.C., enclosing 1s. 3d. It will give you all the information you need. Let me know if I can help further.

A Month's Tour in Switzerland (Nurse H.). First about luggage. No; the ordinary portmanteau will not go under the seat, and you cannot manage *comfortably* with hand luggage for so long a time, but if circumstances make it necessary buy a square Japanese basket, about 20 inches long, cost perhaps 3s. It will expand, and is held by straps with a handle. Supplement this by a bag of the same kind which shuts with a loop and small peg, and can be secured by a padlock. If you take a Cook's circular ticket you are allowed 56 lb. of luggage free, except in Italy. I do not know the pension you speak of, but should guess it to be the Pension de la Croix Fédérale. I should advise your staying as long as possible at Chamonix or Zermatt and Lausanne, and not try to see too many places; you see nothing thoroughly in only two or three days' residence, and constant moves are a heavy task on a slender purse.

Addresses in Bruges (F. M.). If they have room you would be very comfortable at the Hôtel Panier d'Or, which is in the Market Place. I think terms would not exceed 6 francs each, as there are several of you; indeed, perhaps even a less sum might be arranged. Still cheaper, but not so amusing, Mrs. Dear, Pension Internationale, 4 Rue Anglaise; or Mme. Redlich, 30 Rue du Vieux Sac. The private address sent to my other correspondent would not be so suitable. I envy you your first visit to Bruges.

### Echoes from the Outside World.

### The King and Queen at Liverpool.

On Tuesday morning the King and Queen left London for Liverpool, where his Majesty laid the foundation stone of the new Cathedral. The festivities in the city commenced on Saturday, and on Monday the streets were crowded with people viewing the decorations, which were on a most lavish scale. Upon their arrival in Liverpool, where the day had been proclaimed a Bank Holiday, their Majesties were received by the Lord Mayor, and proceeded to the Town Hall amid the cheers of the crowds who lined the streets. After luncheon, addresses were presented, and the King in reply to that of the Corporation, said he was convinced that the Cathedral would minister to the spiritual needs of their great community. Their Majesties then proceeded to the site of the Cathedral, and the King laid the foundation stone. The Cathedral is to exceed in area that of any other in England, and will cover about 90,000 square feet. It will be 584 feet long, and the nave will be 116 feet high. The building, when complete, will accommodate 8,000 people. After the service their Majesties drove to the Prince's landing-stage where the Royal yacht was moored. The yacht left at 5 30 for Swansea, but owing to the prevalence of a gale in the Irish Channel the King and Queen landed at Holyhead and proceeded by rail to South Wales.

### The Queen in East London.

The Queen and Princess Victoria visited the East End of London last week and received a most enthusiastic welcome. Her Majesty had previously announced that she would attend the Flower Show at the People's Palace, but learning that on the same day the Parish Window Garden Society's Show and a bazaar was to be held in the rectory grounds of St. George's-in-the-East, and hearing from the rector that her presence at the Palace would prove a powerful counter-attraction, she kindly offered to be present at both displays. Outside St. George's the announcement, in large red letters, that "The Queen is Coming" created no little excitement, and led to the assemblage of an enormous crowd. Queen Alexandra made a comprehensive tour of the stalls, one of which was completely stocked with flowers which had been sent from Windsor by her Majesty's commands. The Royal party remained in the grounds over 20 minutes, and then drove on to the People's Palace, where she was escorted through the building by the Duke of Fife and Mr. West. In the Winter Garden the juvenile members of the East London Horticultural Society, 650 in number, had assembled and sang a verse of the National Anthem. They were evidently much pleased that the Queen should inspect the flowers which they had grown under such difficult conditions, many of the blooms looking very healthy and strong.

### The Prince and Princess of Wales.

The Prince of Wales presided on Monday at the annual meetings of the Associated Board of the Royal Academy of Music and the Royal College of Music, both of which were held at Marlborough House. His Royal Highness, who on each occasion officiated in his capacity of president, distributed the medals gained by the candidates at the respective examinations, and congratulated the Board of the Academy and the Corporation of the College on the satisfactory record of the past year. On Saturday the Prince of Wales, who was accompanied by the Princess, visited St. Pancras in order to lay the foundation-stone of the new buildings of the Working Men's College. The new buildings will occupy a commanding and open site at the corner of Crowndale Road and Camden Street. There will be a hall to accommodate 250 persons, offices for administrative purposes, with common-room, club-room, and gymnasium for the students, a library fitted for 10,000 books, a museum, and a number of class-rooms. Altogether, there is teaching space for 700 students.

### The War in the Far East.

A report that last week the Japanese had 30,000 casualties near Port Arthur on July 10th or 11th has been officially contradicted by the Japanese Imperial Headquarters Staff, who declare that not a shot was fired on either date. It is still, however, maintained in St. Petersburg that the Japanese have suffered heavy losses, while the Russian casualties are said to have amounted to 5,500. Early on Sunday morning 20,000 Russians attacked the Japanese on the Mo-tien-ling Pass. The fight lasted 15 hours, and according to a despatch from General Kuropatkin it was of a most serious character, the Russians having over 1,000 casualties. The Japanese estimate the Russian losses at 2,000 and their own at 300. A steamer belonging to the Peninsular and Oriental Company has been seized by the Russians and ordered to Sebastopol. A Japanese destroyer has seized a junk containing the mails from Port Arthur to Chifu. The letters bearing on the naval and military conditions of the port were taken possession of, and much valuable information obtained.

### Death of Paul Kruger.

Ex-President Kruger died last week at Clarens, Switzerland, of pneumonia complicated by heart disease. Before his death he expressed a desire to be buried in the Transvaal. A petition to that effect was at once addressed by his family to the British Government, and consent was given to the ceremony taking place in Pretoria. The body will probably be laid to rest in the special cemetery set apart for Presidents of the late Republic, where Pretorius and Burgers are buried. Paul Kruger—who was nearly 80 years of age at the time of his death—was born in Cape Colony, and when he was 11 he and his family joined in the trek led by Potgieter from the Orange River, and took possession of the territory between the rivers Wet and Vaal. Here, though so young, he joined in the fights between the Zulus and Dutch, and was once in a massacre of settlers when only two boys beside himself escaped. In 1883 he became President of the Transvaal. He was always very anti-English in his ideas, and, although he accepted office and salary under the British Government, he readily profited by the opportunity to inflame local feeling against Great Britain, and would accept no overtures from her. His refusal of common rights to the always increasing alien population grew more oppressive year by year, and at last in October 9th, 1899, believing himself strong enough to impose his will upon England, he delivered an ultimatum declaring war upon Great Britain in 48 hours. During that war he seldom, if ever, appeared in the field, but remained quietly smoking his pipe in Pretoria. When he found that the British were advancing he escaped to Europe. He was twice married: his first wife had one son, who died.

### More Changes in the Colonial Service.

The King has approved the appointment of Sir William MacGregor as Governor and Commander-in-Chief of Newfoundland, and of Sir Gilbert Thomas Carter as Governor of Barbados. Sir William MacGregor, who is at present Governor of Lagos, is a distinguished medical man. He recently arrived in this country with the Alake of Abeokuta, was administrator of British New Guinea for seven years, and Lieutenant-Governor for three years. He is the possessor of the gold medal for saving life at sea. Sir Gilbert Carter is at present Governor of the Bahamas, and has also been Governor of Lagos. He began his career in the Navy, and entered the Colonial Service in 1875 as private secretary to the Governor of the Leeward Islands. Both Governors are 56.

### A Book and its Story.

### INFATUATION.*

The romance of a woman of forty married to a man who is young and handsome, with a reputation that had earned for him the name, as far as women were concerned, of the "Fool-killer," is the motive of Lucas Cleave's admirably written novel. Lady Marchmont is a radiant and beautiful woman of the world, a widow who has retained her dignity and her heart in spite of the offers of many adorers who aspired to succeed her late husband. She had not, until after her only daughter's marriage, seriously thought of marrying again. There had been a past episode in her early married life in which a friend of her husband's had figured conspicuously. After Lord Marchmont's death he returned from abroad to fulfil the promise made to him to marry her if she would have him. But, although she had not had a happy married life through the want of mutual understanding and sympathy between her husband and herself, she felt it more just to his memory not to marry Sir Hepworth Heatherby, for whom she had no longer any warm feeling.

The book begins on the wedding day of the daughter, Avril, so-called, as her mother explained, "because she came in the month following March." Avril, as is so frequently the case with girls who have a brilliant mother, was an exceedingly matter-of-fact daughter, of whom Lady Marchmont stood somewhat in awe. On the evening of Avril's wedding day her thoughts were divided between the sense of desolation, which their twenty years together made very apparent, and a sense of relief that at last she was free to live her own life undisturbed by the criticisms and restraints which Avril's presence put upon many small matters that were a joy to her. "People told her it was ridiculous to let a child domineer over her and make rules for her life; but then they did not know that Lady Marchmont's idiosyncrasy was a belief in the moral law of punishment, to which she even sacrificed her belief in justice and mercy. . . . Every time she obeyed Avril it seemed to her that she redeemed the past. Avril was so like her father. . . ." Looking back on the twenty years of Avril's life, it seemed to Lady Marchmont that they had been twenty years of repression; and yet she had said to herself, "I can understand how slaves feel when they are given their freedom, and don't want it." Joseph, the poodle, noticed at once when Lady Marchmont began to cry, and he jumped up on the sofa beside her. Instinctively Lady Marchmont pushed him down; Avril had never allowed him on the sofa. But Joseph meant to put his foot down at once, the one with the tiny bangle on it, and to try and show his beloved "*que nous avous changé tout cela.*" He meant always to sit on the sofa now—openly, defiantly. With the air of a dog who means to live an honest life thenceforward, he jumped up again beside her and laid his paw on her dress, the grey silk which Avril had said was the right kind of dress for her mother to wear for her wedding." Avril had ordered the gown as she had ordered everything else for her wedding. The cake, the bridesmaids' dresses, the decorations for the church, the trousseau—and everything had been so like her—a little heavy, gorgeous and splendid enough, but lacking grace. "The expression of a soul possessed of the wisdom of goodness but lacking in the fragrance of romance, in the diaphanous delicacy of tenderness." "What a hateful dress! What an awful dress!" Lady Marchmont had murmured to herself. And the "bulbous" in her make-up was exactly the thing that had delighted her son-in-law, who was a Dutchman with a passion for tulips. "It was the 'bulbous' in her husband's nature that had always bewildered her." Her reflections end in arriving at the conclusion that "There really seems no excuse for me to go on living. When one's children marry, a carriage ought to come and drive one away." And afterwards she became conscious that she was growing fainter in her loyalty to Avril since she had gone away. She had made it a custom to praise her, but when she realised the rigidity of Avril she wondered after all if she would have been better if she had emulated this characteristic instead of allowing herself to be a flower whose nature was to stretch wildly and aimlessly over everything in tangled luxuriance; and instead, she had been cut and trimmed by time, and of late years, trimmed by Avril's knife on to alien growths from which her spirit recoiled; something deep down in her heart told her that life had not played her fair, that she had been robbed of the inheritance of a woman—the right to love and be loved."

Standing there, "even in that awful grey dress," she looked supremely lovely in the eyes of her youthful lover, who came in the evening with theatre tickets after having seen off the bride and bridegroom. They had been a quarter of an hour too early, which was what Lady Marchmont had expected. It seemed to her that all their life they had been too early for trains, for Avril declared that all the discomfort in life arose from the want in people to see to things in time. But there had been a mistake about a reserved carriage which was disconcerting also, but Rupert Cunliffe had arranged matters satisfactorily. "It was so like Rupert Cunliffe to go to the station and see to things." And now the two were alone. No thought of Rupert Cunliffe as a lover had ever crossed Lady Marchmont's mind. "You will come and see Duse?" "I thought you would be feeling a little dull," he said. "How nice of you, but I have just been asking myself whether a convent or the country would be the best place for me. I am afraid of my liberty. I feel like a pivot that has got loose." "Why, now your day has come at last; thank God for creating the Dutchman." Lady Marchmont laughed and then sighed. "My day was over long ago. Doesn't this dress proclaim me to be a mother-in-law? The next step will be to become a grandmother." "Do you remember that book, 'The Baby's Grandmother'; you always remind me of the grandmother." Lady Marchmont laughed again. She was too loyal to say Avril always reminded her of the daughter. "You don't know how awfully young you look." "Do you know I am old enough to be your mother, I was forty yesterday." "I wish you would not say such things anyhow, you are much younger than Avril, for instance." "Oh, Avril; she was born old, I think. Her father was much older than I was. I think such marriages are a mistake: there is only one thing worse, and that is for a woman to be older than a man." "I don't think so. I think it is only a woman of a certain age that understands a man. I couldn't marry a *jeune fille* if you paid me." Lady Marchmont finally, after more than one rejection, accepts Rupert Cunliffe. A letter from Avril, written after the announcement of her mother's wedding, explained her feeling about the matter. "I shouldn't have thought it possible that you could have been so foolish," she wrote, "and of all men in the world Mr. Cunliffe! If you had married a nice old man of your own age; but a boy like that, who is only after your money! It is ridiculous, and you cannot expect Heidebrand or myself to be present at the wedding." How the marriage turned out is not for us to say. Our readers will find the result of the experiment told in the book itself.

* The Fool-killer. By Lucas Cleave. Fisher Unwin. 6s.

### Notes and Queries.

**FOR REGULATIONS SEE PAGE 143.**

*Nursing Lepers.*

(121) (1) Can you give me an address in England where I can apply for information as to the nursing of lepers? (2) Is there any demand for such nurses?

(1) Perhaps the Royal Victoria and Albert Dock Hospital (branch of the Seamen's Hospital, Greenwich) could help you, or the Mission to Lepers in India and the East, 17 Greenhill Place, Edinburgh. (2) None in England and little abroad.

*New York Training.*

(122) I wish to go to New York to train. (1) Where can I apply for rules? (2) And can I gain an engagement before crossing?—*E. A. B.*

(1) Consult "The Nursing Profession: How and Where to Train" for list of New York hospitals, then write for particulars. (2) We are afraid not.

*Holiday.*

(123) I am leaving at the end of 14 months' service and am entitled to a holiday at the end of the year. Can I claim the same or salary in lieu thereof?—*Alice J. H.*

As holidays are given to enable nurses to continue their work, and you are giving up yours, we do not think that your claim would be entertained. You could not ask for salary instead.

*Fever Work.*

(124) I am anxious to obtain a post in the Tooting Fever Hospital; would that be possible without general training? I have had four and a half years' fever experience in all branches of the work including small-pox.—*H.N.B.*

Write to the Matron for all particulars.

*Electric Sol Treatment.*

(125) I shall be much obliged if you will kindly tell me where a course of lectures on, or training in, the Electric Sol treatment can be obtained, what the cost would be, and the length of time occupied by such a course?—*Nurse W.*, L O S.

An institution has been opened at 36 Edgware Road, W., for special treatments; probably the manager could give the required information.

*Sanatoria.*

(126) I should be grateful for the addresses of one or two sanatoria for the treatment of consumption situated in Surrey or Sussex.—*M.R.*

The Crooksbury Sanatorium, Crooksbury Ridges, Farnham, Surrey, or the Whitmead Hill Sanatorium, Tilford, near Farnham.

Will you kindly tell me of a sanatorium into which I could get a poor girl who could only afford to pay about 5s. a week?—*Nurse A.M.B.*

The National Sanatorium for Consumption and Diseases of the Chest, Bournemouth. The charges are 7s. 6d. a week with a governor's nomination.

*Mouth Shut.*

(127) Can you kindly tell me if there is any appliance for keeping the mouth shut whilst asleep, and where I can obtain it?—*M.B.*

Do not trust to appliances; consult a medical man as you should make sure that there is no obstruction to breathing freely, such adenoids.

*Maternity Fee.*

(128) Will you kindly tell me what compensation I can claim from a lady who engaged me for monthly nursing, and who, a short time before I was required, cancelled the engagement in order to get a nurse at a smaller fee?—*Nurse E.*

You had better put the matter into the hands of a solicitor.

**Important Nursing Text-books.**

"The Nursing Profession: How and Where to Train." 2s. net; 2s. 4d. post free.

"The Nurses' Dictionary." By Honnor Morten. 2s. post free.

"Nursing: a Complete Text-book of Medical, Surgical, and Monthly Nursing." By Percy Lewis, M.D. 3s. 6d., post free.

"Elementary Physiology for Nurses." By C. F. Marshall, M.D. 2s. post free.

"A Complete Handbook of Midwifery for Nurses." By J. K. Watson, M.D. 6s. net; 6s. 4d. post free.

The above works are obtainable of all booksellers, or direct from The Scientific Press, Limited, 28 & 29 Southampton Street, Strand, London, W.C.

### For Reading to the Sick.

"HE SHALL SUSTAIN THEE."

Thy burden is God's gift,
And it will make the bearer calm and strong;
Yet, lest it press too heavily and long,
He says, "Cast it on Me,
And it shall easy be."

And those who heed His voice,
And seek to give it back in trustful prayer,
Have quiet hearts that never can despair,
And hope lights up the way
Upon the darkest day.

It is the lonely load
That crushes out the light and life of heaven;
But borne with Him, the soul restored, forgiven,
Sings out through all the days
Her joy and God's high praise.

*Anon.*

"Cast thy burden on the Lord and he shall sustain Thee." Our burden, whatever it is, is God's "gift," and has a divine blessing in it for us, if we take it up in faith, in love. . . . We are not promised that the burden shall be lifted away from our shoulder, or that it shall be borne for us, but that we shall be sustained in carrying it ourselves. If it is God's gift, it is His will that we should keep it, at least for the time. There is some blessing in it for us, and it would not be kindness to us for God to take it away, even at our earnest pleading. It is part of our life, and is essential to our best growth.—*Dr. Miller.*

No burden that our brother can bear is nothing to us; his burden is our burden, as our burden is the burden of our and his merciful Saviour. To sympathise with his repentance may be the means which God has provided to bring us to a sense of our own sin.—*Isaac Williams.*

The crosses which we make for ourselves by over-anxiety as to the future are not heaven-sent crosses. The crosses actually laid upon us always bring their own special grace and consequent comfort with them; we see the hand of God when it is laid upon us. But the crosses wrought by anxious forebodings are altogether beyond God's dispensations; we meet them without the special grace adapted to the need—nay, rather in a faithless spirit, which precludes grace. And so everything seems hard and unendurable; . . . "Sufficient unto the day is the evil thereof," our Lord has said; and the evil of each day becomes good if we leave it to God. Let us shut our eyes to that which God hides from us in the hidden depths of His wisdom. Let us worship without seeing; let us be silent, and lie still.

*Fenelon.*

Within every healing shadow is God Himself; and so, though it seem to be a shadow of the sorest sorrow and pain, yet will it lift me upward and lead me into the light.

*Robert Collyer.*

I do not ask my cross to understand,
My way to see;
Better in darkness just to feel thy hand,
And follow Thee.

*Adelaide A. Procter.*

